# Case report: Ultrasound-Assisted endovascular therapy for carotid artery floating thrombus

**DOI:** 10.3389/fcvm.2022.961760

**Published:** 2022-09-14

**Authors:** Peng Wang, Zhenzhen Wang, Jie Pan, Kefeng Lu, Litao Sun, Yu Geng

**Affiliations:** ^1^Department of Neurology, Center for Rehabilitation Medicine, Zhejiang Provincial People's Hospital, Affiliated People's Hospital of Hangzhou Medical College, Hangzhou, China; ^2^Ultrasound Medicine, Zhejiang Provincial People's Hospital, Affiliated People's Hospital of Hangzhou Medical College, Hangzhou, China

**Keywords:** free-floating thrombus of the carotid, endovascular thrombectomy, carotid endarterectomy, carotid angioplasty and stenting, ultrasound-guided intervention

## Abstract

**Background:**

Carotid free-floating thrombus (CFFT) is a rare but sometimes emergent condition. There has been controversy over the optimal treatment strategy. Emerging evidence suggests that endovascular thrombectomy (EVT) may be an alternative to surgery. Accurate alignment of the aspiration catheter and thrombus during EVT is critical but has, so far, remained unresolved.

**Case summary:**

This is a rare case of CFFT presenting with acute right-sided facial droop and moderate dysarthria in a 77-year-old man. He was in sinus rhythm with a blood pressure of 110/82 mmHg. Both non-contrast CT (NCCT) and head CT angiography (CTA) were unremarkable, while whole-brain CT perfusion (WB-CTP) suggested left hemisphere core infarction. Delayed imaging of the left internal carotid system by 4D-CTA suggested severe proximal obstructive disease, as confirmed by carotid CTA and ultrasonography. The initial two aspirations under DSA were invalid due to the challenging anatomical angle between the thrombus and the catheter. The success of CFFT removal was achieved with a pressure-assisted ultrasound-guided approach that helps to compress the catheter tip toward the thrombus.

**Conclusion:**

We innovatively report a successful ultrasound-guided EVT for CFFT. Ultrasound assistance can provide quick and effective guidance and may guide tailored aspirations during EVT.

## Introduction

Carotid free-floating thrombus (CFFT) has been reported as a rare entity but may present with emergent symptoms (1). In cases that are refractory to anticoagulation therapy, carotid endarterectomy (CEA) or carotid angioplasty and stenting (CAS) may be effective. However, there has been controversy regarding the optimal treatment strategy (1). Evidence suggests that endovascular thrombectomy (EVT) is emerging as an alternative to CFFT surgery and may yield good long-term outcomes (2, 3). Notably, accurate alignment of the aspiration catheter to the thrombus is critical during EVT, which has not been addressed to date. We innovatively report the successful intraoperative pressure-assisted ultrasound-guided direct aspiration of CFFT in the presence of technical obstacles caused by the anatomical angle between the plaque and catheter tip.

## Case description

A 77-year-old Chinese male with only a medical history of hypertension was transferred to our hospital with acute right-sided facial droop and moderate dysarthria. About 16 h before admission, he experienced a gradual onset of weakness and numbness of the right upper extremity, followed by slurring of speech. On admission, he was in sinus rhythm with a blood pressure of 110/82 mmHg. His National Institute of Health Stroke Scale (NIHSS) was 6, Modified Rankin Scale (MRS) was 3, and Water-Swallow Test Score was 2. Only D-dimer (1,030 μg/L) and prothrombin time (12.5 s) were elevated in laboratory tests. He is a non-smoker and has no other medical history or alcohol addiction. There was no evidence of primary or acquired hypercoagulability, such as cancer or thrombotic disorders.

## Diagnostic assessment

**Figure 1:**
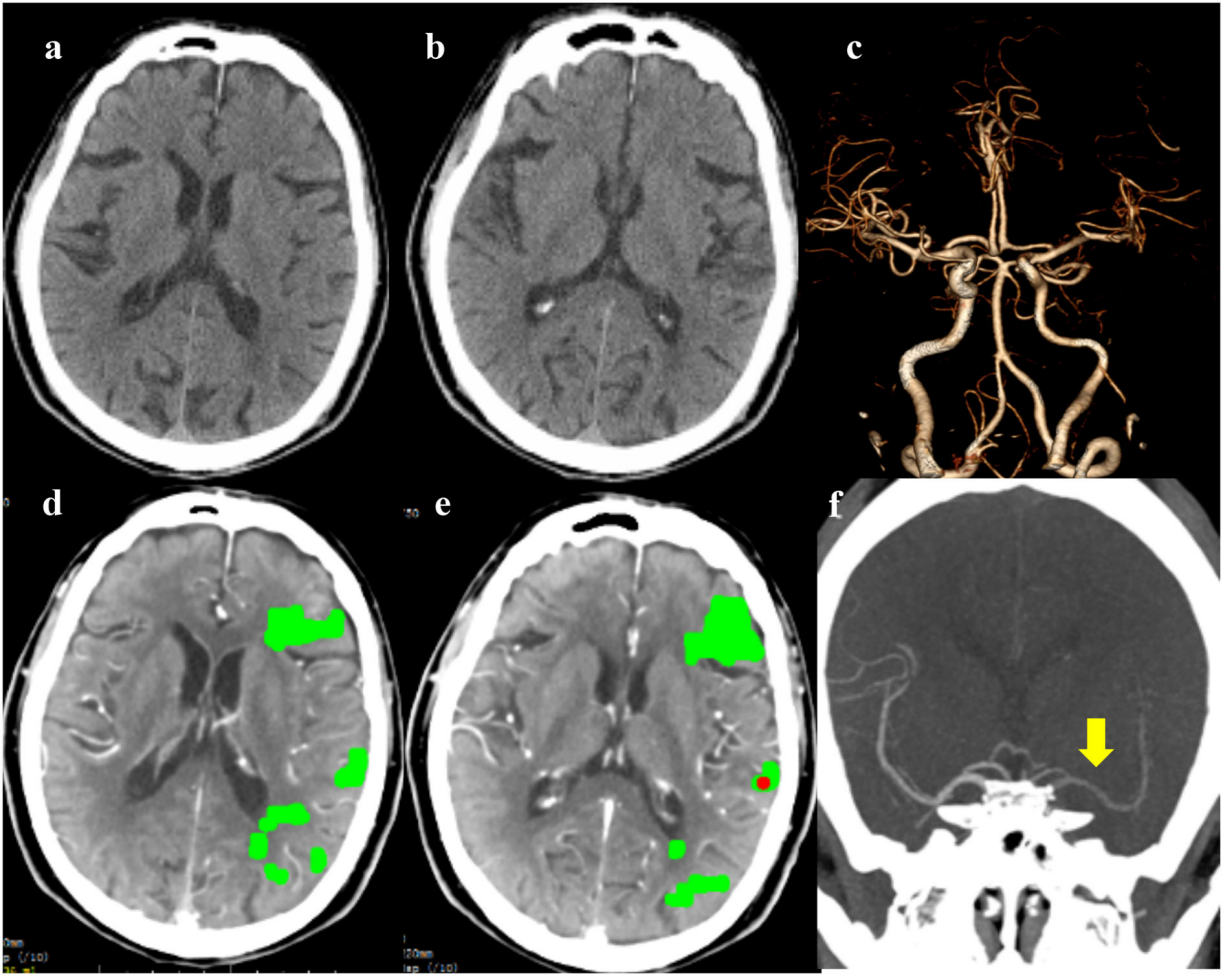
The emergency non-contrast CT (NCCT) **(a,b)** and cranial CT angiography (CTA) **(c)** did not show obvious cranial abnormalities; whole-brain CT perfusion (WB-CTP) indicated the left hemisphere core infarction **(d,e)**; The 4D-CTA reconstruction showed delayed imaging of the left middle cerebral artery [**(f)**, yellow arrow].

**Figure 2:**
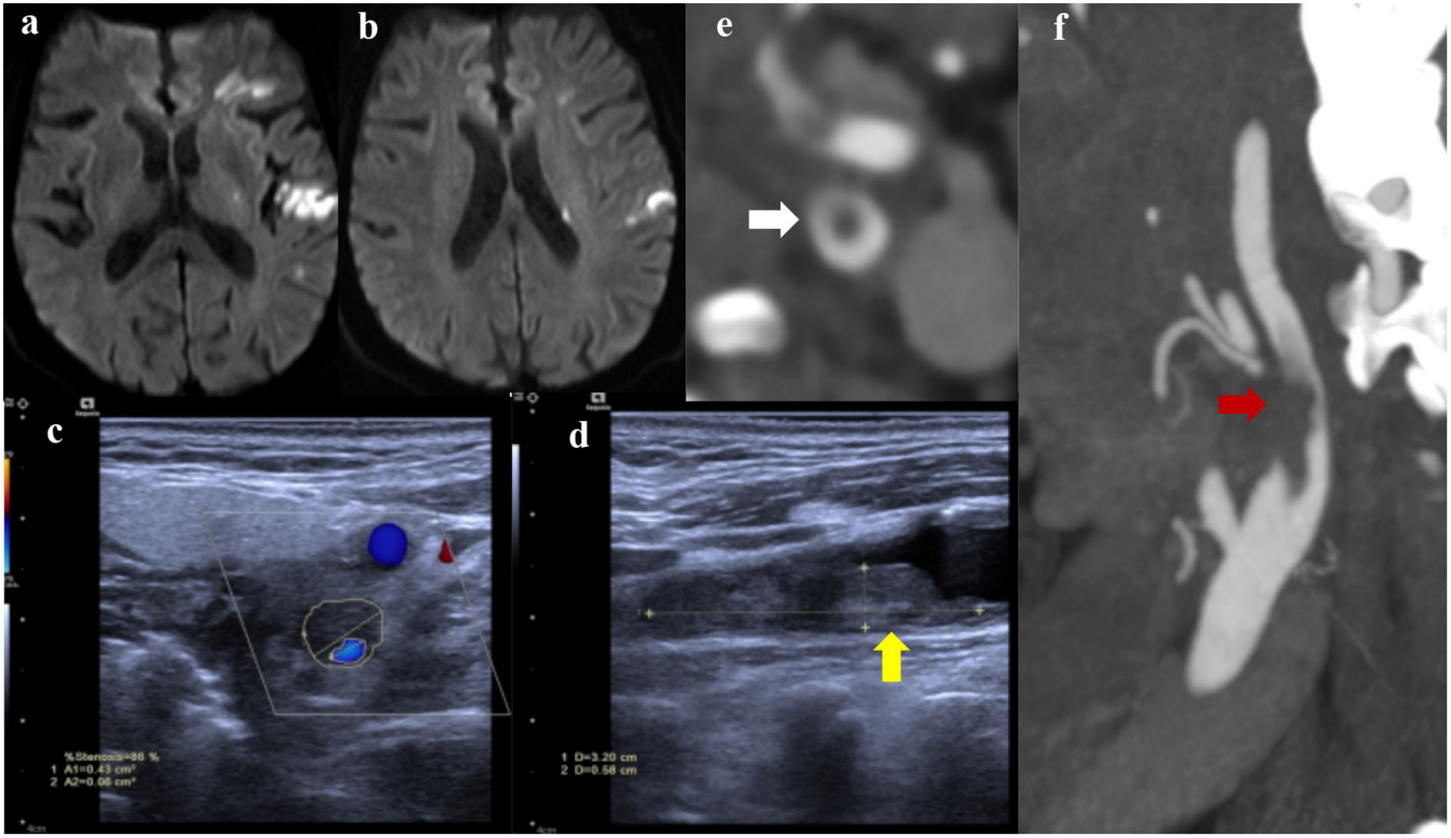
Diffusion-weighted imaging (DWI) showed multiple infarctions in the left hemisphere **(a,b)**; Carotid ultrasound **(c,d)** indicated a culprit plaque with local floating thrombus (yellow arrow), resulting in obvious stenosis of the left internal carotid artery; carotid CTA **(e,f)** presented the floating thrombus (red arrow) with a typical cross-sectional “donut sign” (white arrow).

There were no obvious abnormalities in emergency NCCT and CTA, and the Alberta Stroke Program Early CT Score (Aspects) was 10 points. However, the WB-CTP indicated the left hemisphere core infarction was 1 ml with a penumbra zone of 35 ml (MIStar, Apollo Medical Imaging Technology, Melbourne, Australia). The 4D-CTA reconstruction showed delayed imaging of the left internal carotid system, suggesting severe proximal obstructive disease (Figure 1). During hospitalization, daily prescriptions included enteric-coated aspirin 100 mg in combination with clopidogrel 75 mg and atorvastatin 40 mg. Diffusion-weighted imaging (DWI) showed multiple infarctions in the left hemisphere, and carotid ultrasound showed 85% stenosis of the left internal carotid artery with local floating thrombus. Two days after admission, there was no obvious change in the ultrasound review, and the carotid CTA presented a typical “donut sign,” indicating a CFFT rather than only a vulnerable plaque (Figure 2).

**Figure 3:**
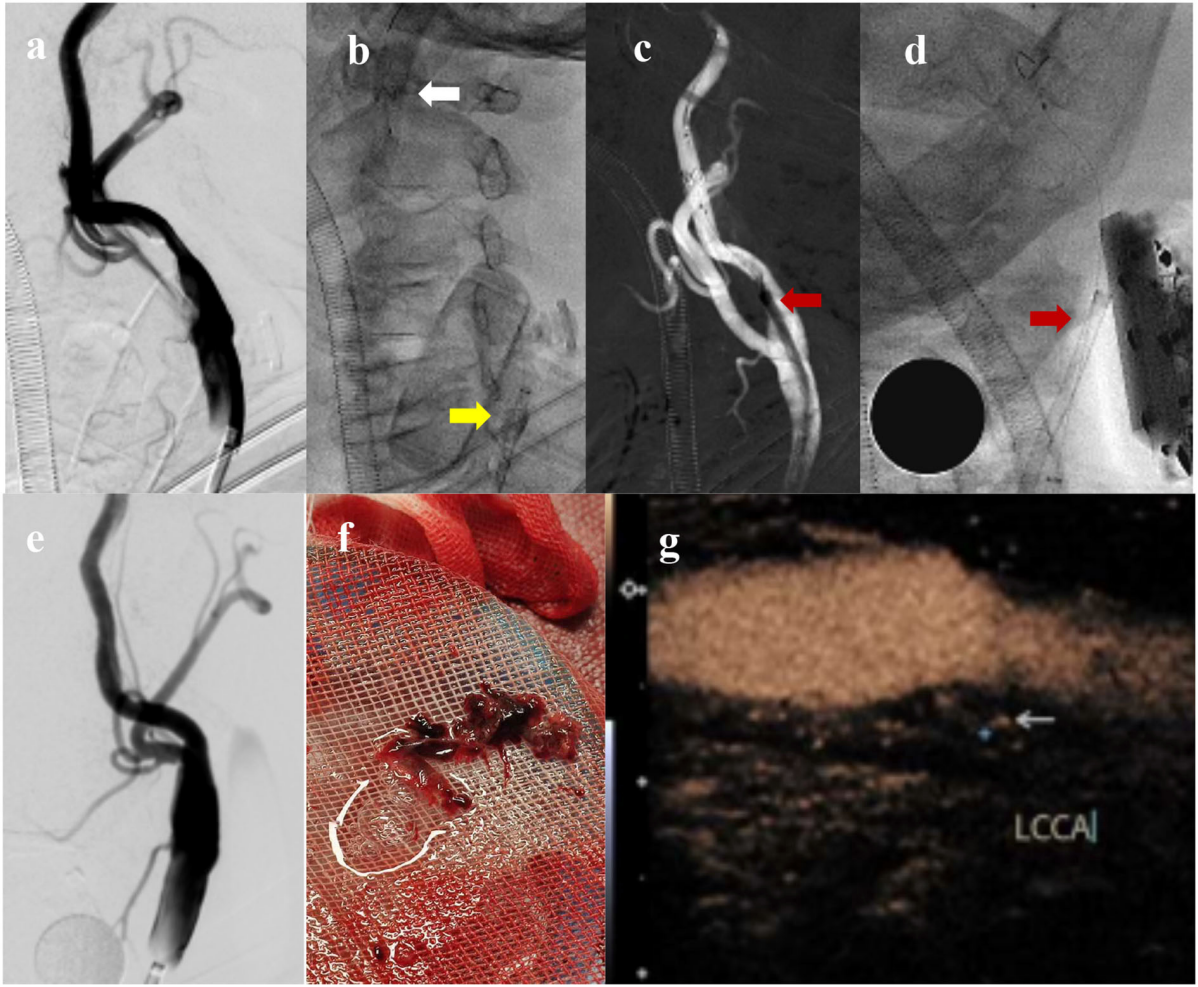
Carotid DSA before aspiration **(a)**; The FlowGate^2^ balloon guide catheter (yellow arrow) is placed in the left common carotid artery and the Emboshield NAV6 embolic protection system (white arrow) is placed in the distal segment of the internal carotid artery **(b)**; The Mach1 guide catheter (red arrow) is inserted to aspirate CFFT **(c)**; Ultrasound probe depressing Mach1 tip (red arrow) to face the thrombus **(d)**; Carotid DSA after aspiration **(e)**; Massive thrombus has been aspirated **(f)**; Postoperative contrast-enhanced ultrasound showed unstable plaque with no evidence of CFFT **(g)**.

**Figure 4:**
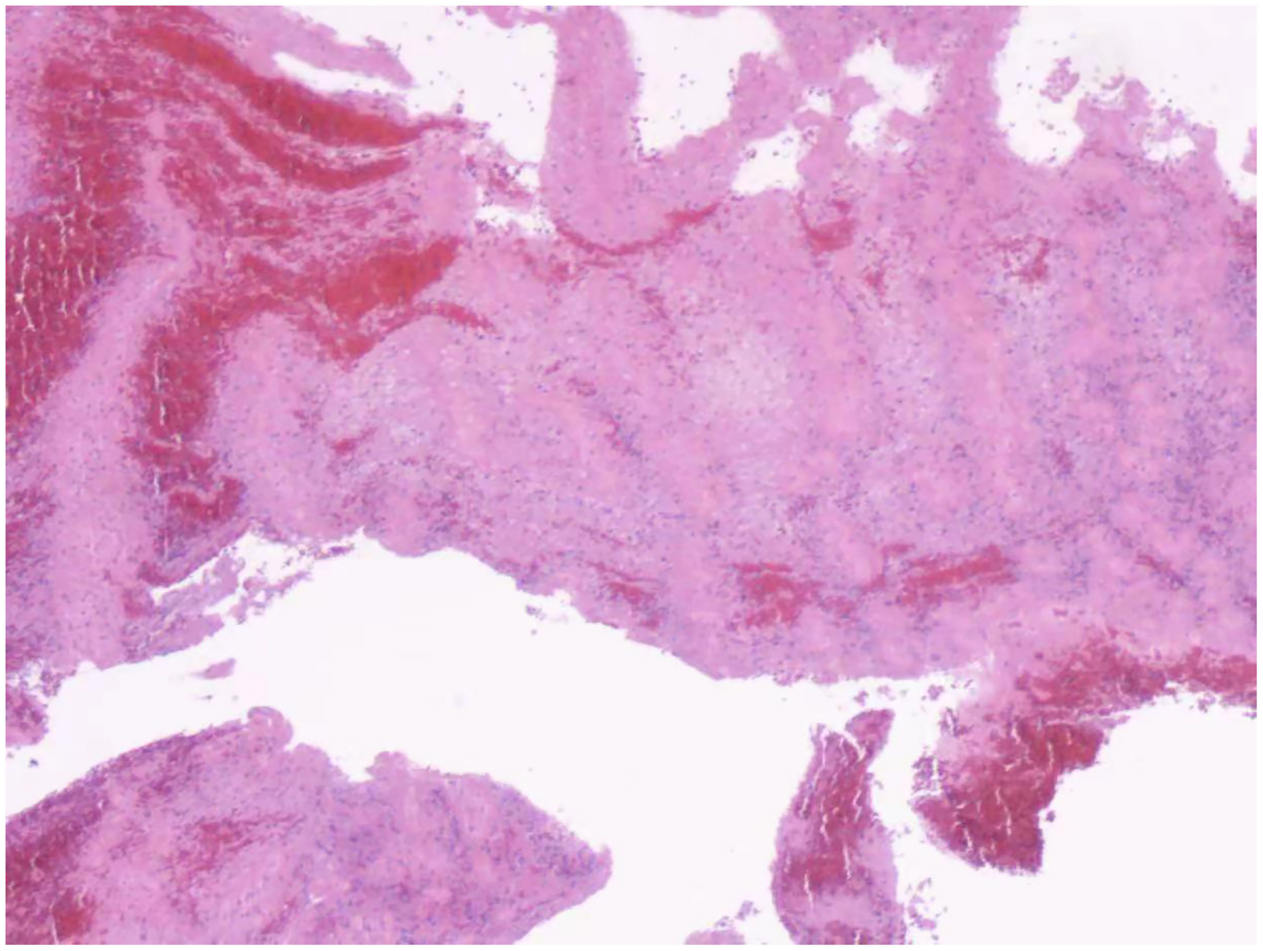
Hematoxylin and eosin stain presented a fresh thrombus that was rich in red blood cells and platelets.

Considering the onset-to-admission time (16 h) and evidence of CFFT on CTA, medical therapy may be invalid, and, in the shortest time, EVT was recommended according to the guidelines for the treatment of acute ischemic stroke with large vessel occlusion (4). A bilateral femoral artery approach was adopted to insert the 8F arterial sheath. One 8F balloon guide catheter (FlowGate^2^ Balloon Guide Catheter, Stryker Neurovascular, USA) entered the left common carotid artery. Afterward, a Nav6 umbrella (Emboshield NAV6, Abbott Vascular, USA) was inserted through the catheter and placed in the distal segment of the internal carotid artery further than the floating thrombus. This help prevents distal embolization events from thrombus falling during the operation. Another 8F guide catheter (Mach1, Boston Scientific, USA) was inserted to aspirate the CFFT. However, only a small amount of thrombus was aspirated during the first two local aspirations. The Mach1 could not accurately head to the thrombus during aspiration with only the longitudinal imaging information of the thrombus identified under DSA. Therefore, an ultrasound-guided approach was initiated to visualize the location of the CFFT. The ultrasound illustrated the reason for the first two invalid aspirations by showing that the Mach1 could not head toward the thrombus due to the vascular anatomy and residual eccentric lumen. Based on the transverse view of the internal carotid artery, while viewing the Mach1 tip above the thrombus, we gently pressed the probe (1 mm/s) to reduce the distance between Mach1 tip and the thrombus. During the procedure, real-time ultrasound recorded the aspiration process and the vibrating performance of the CFFT (Supplementary Video 1), and color Doppler flow imaging confirmed CFFT removal after a single ultrasound-guided aspiration (Supplementary Video 2). We saw the thrombus aspirated from Mach1, and repeated DSA also confirmed the removal of the thrombus. There was no escape thrombus in the protective umbrella during surgery, and post-operative DWI showed no evidence of a new infarct. On the next day, contrast-enhanced ultrasound (CEUS) showed only an unstable plaque in the region of CFFT (Figure 3). Pathological examination revealed fresh thrombi, rich in red blood cells and platelets (Figure 4). During the initial 2-week follow-up, the patient was asymptomatic and remained clinically stable. Contrast-enhanced ultrasonography of the carotid artery is recommended after 2 weeks to see if further anticoagulation is required.

## Discussion

The ischemic event may have resulted from the rupture of vulnerable atherosclerotic plaques of the internal carotid artery in the patient's hypertensive setting. CFFT was reported as early as 1905 (5), and subsequent reports were mostly found in surgical operations (6). With the improvement of CTA diagnostic technology, the incidence rate has increased from 0.4 to 1.5% based on catheter angiography to 3.2%, especially owing to the proposed “donut sign” (7). A literature review has emphasized the importance of the “donut sign” and discussed the etiology and treatment of CFFT, pointing out that it was mostly atherosclerotic disease (82%), and recommending heparin combined with an antiplatelet drug for treatment (8). However, there is still a 7.5% stroke recurrence rate and a 3.5% mortality rate, with a median event time of 2 days (IQR 1–8 days). Therefore, early identification of medication refractory and effective selection of alternative treatment options are extremely important.

Some scholars have reported that CEA is safe and effective in the treatment of CFFT (9). There are also reports of CAS in the treatment of CFFT (10). Recently, with the development of endovascular treatment of acute ischemic stroke with intracranial large artery occlusion and the introduction of minimally invasive and non-implantation concepts, more centers begin to try mechanical thrombectomy to treat CFFT (11). However, procedures of direct aspiration are extremely rare (12); there are limited reports regarding an ultrasound-guided approach to resolving CFFT. Otawa et al. (13) and Giragani et al. (14) have reported that ultrasonography helped localize CFFT during endovascular therapy. In our case, the major technical barriers include the small diameter of the catheter and the limited angle of the catheter tip, resulting in it being extremely difficult to accurately head toward the body of the thrombus. Thus, we innovatively performed a pressure-assisted ultrasound-guided aspiration. Our experience highlights that the carotid ultrasound may provide a simple and effective approach to the accurate aspiration of CFFT, which is recommended in the management of such difficult cases during the EVT treatment of CFFT.

## Data availability statement

The original contributions presented in the study are included in the article/Supplementary material, further inquiries can be directed to the corresponding authors.

## Ethics statement

The studies involving human participants were reviewed and approved by Zhejiang Provincial People's Hospital, Affiliated People's Hospital of Hangzhou Medical College. The patients/participants provided their written informed consent to participate in this study and for the publication of this case report.

## Author contributions

PW performed the EVT and wrote the main part of the manuscript. ZW performed the ultrasound scan during the intervention and revised the manuscript. JP and KL were assistants during the operation. LS and YG were directors of this research and program. All authors contributed to the article and approved the submitted version.

## Funding

ZW and LS received grants from the National Natural Science Foundation of China (82001841 and 82071929). These funds were received for the research work and open access publication fees.

## Conflict of interest

The authors declare that the research was conducted in the absence of any commercial or financial relationships that could be construed as a potential conflict of interest.

## Publisher's note

All claims expressed in this article are solely those of the authors and do not necessarily represent those of their affiliated organizations, or those of the publisher, the editors and the reviewers. Any product that may be evaluated in this article, or claim that may be made by its manufacturer, is not guaranteed or endorsed by the publisher.

## Supplementary material

Supplementary Video 1 Ultrasound-guided aspiration of CFFT. On transverse view of the left internal carotid artery, a Mach1 guide catheter was placed facing the plaque under gentle pressure from the ultrasound probe. The thrombus was vibrating when the aspiration was initiated.

Supplementary Video 2 Color Doppler flow imaging confirmed the removal of the CFFT.
